# Editorial: Biopharming, volume II: new plant breeding technologies for metabolic engineering or recombinant proteins production

**DOI:** 10.3389/fpls.2023.1231422

**Published:** 2023-06-27

**Authors:** Giuseppe Dionisio, Jitendra Kumar Thakur, Tanushri Kaul

**Affiliations:** ^1^ Aarhus University, Department of Agroecology, Crop Biotechnology and Genetics Section, AU-Flakkebjerg, Slagelse, Denmark; ^2^ Crop Health Section, Department of Agroecology, Faculty of Technical Sciences, Aarhus University, Slagelse, Denmark; ^3^ International Centre for Genetic Engineering and Biotechnology (India), New Delhi, India

**Keywords:** plant biotechnology, new breeding technologies (NBTs), CRISPR/Cas9, bio-pharmaceuticals, recombinant antigen, recombinant antibodies, plant metabolic engineering, recombinant protein

In the Plant Biotechnology section of Frontiers in Plant Science, many articles are related to the production of recombinant proteins in plants, also called Biopharming. The first Research Topic about Biopharming introduces arguments regarding engineering of crops for the bio-production of recombinant proteins (i.e., enzymes or antibodies).

Plants need to be genetically engineered using Agrobacterium-mediated or protoplast transformation prior to being used as bio-factories producing pharmaceutically active compounds (metabolites), or in this case, to form bio-platforms for producing pharmaceutical proteins with reduced proteolysis and purification costs.

The use of “New Breeding Techniques” (i. e., CRISPR/Cas9, TALENs, Zinc Finger) to develop new plant traits has been rapidly adapted to engineer (via gene editing) genes regulating specific pathways and deviating or tunneling the metabolic flux of chemical metabolic intermediates toward a specific pathway. Gene editing coupled with traditional overexpression using transgenic (i. e. CaMV-35S promoter driven foreign) genes or cis-genic (own promoter-driven endogenous) genes can create new traits and new crops producing a new set of secondary metabolites targeting human health or industrial interest. The use of protoplast and Cas9/SgRNA (Ribonucleoprotein complex) transformation using electroporation or polyethylene glycol (PEG) has further reduced the need for marker segregation.

However, the production of transgenic proteins has been always the main purpose of biopharming. This Research Topic thus focuses on further implementation of previously described processes that were specifically oriented to optimize the expression of recombinant proteins in plants through smart genetic design of constructs (promoter elements, codon optimization, terminator length), implementation of effective purification tags, or CRISPR/Cas9-mediated abolition of endogenous proteolysis, and finally, reduction of oxidative damage. Besides, if CRISPR/Cas9 is applied to the plant N- or O-type glycosylation system it could reduce the risk of allergenic effects on the biopharmaceuticals proteins produced. Plant proteolysis reduction is also a topic contemplated here and performed using CRISPR/Cas9 aided disruption of annoying proteases, for example, as those performed by Singh et al. to produce proteolysis-free recombinant antibodies in *Nicotiana benthamiana*.

Construct optimization is critical for the correct expression of heterologous proteins in plant cells as most post-translational modifications are present in plants; however, the Kozak sequence and codons need to be optimized. The choice of the promoter, viral or endogenous, containing the desired elements, is also important as well as the 3′ UTR responsible for mRNA stability. As a proof of concept using *N. benthamiana*, Yun et al. have built an artificial transcriptional system for optimally producing high levels of recombinant proteins in tobacco. A complete up to date overview of the different optimization levels for expressing and extracting recombinant proteins in plants has been provided by Coates et al.

Recombinant antibodies, both IgG or IgE, seem to be heterologously expressed in transient plant expression systems (for a maximum of 10 days post infection) at high levels if a geminiviral vector system is used (Bhattacharjee et al.). A transient expression system is always chosen for heterologous proteins that are not targeted to subcellular compartments, which might serve as a safe container before extraction and purification. Upon stable transformation, tagging the heterologous recombinant proteins to be stored safely into endogenous compartments is advisable. This process is often called microencapsulation, and occurs *in vivo* by directly targeting the recombinant protein into protein storage bodies (Hofbauer and Stoger). Grains are often safe compartments for long storage and ER derived prolamin-storage organelles are good storage compartments. A good signal peptide fused to a recombinant protein with a KDEL or the mature 27 kDa γ-zein sequence for C-t targeting represents a good targeting strategy to ER-derived storage bodies.

However, before the coronavirus pandemic spread, tobacco was the preferred plant organism used for producing anti-Covid-19 neutralizing antibodies (Shanmugaraj et al.) or vaccine candidates (Royal et al.). Nevertheless, an increasing amount of modern crop callus systems is also being rapidly developed as biopharming platforms (Gerszberg et al.). Callus or single cell algae represent an alternative to rapid screening and for the stable transformation of heterologous protein production in bioreactors. In this Research Topic we have included the successful production and secretion of functional SARS-CoV-2 spike protein in *Chlamydomonas reinhardtii* by Kiefer et al. Notably, the use of the unicellular green alga *C. reinhardtii* is not new to the biopharming world; however, its use for optimizing expression systems, engineering further proteolysis sites (i.e furin site), secretion signals (Molino et al.) or N-type glycosylation is rather interesting. Further, SARS-CoV-2 spike protein (or the RDB portion) can be an attractive production platform for next round of vaccination against dangerous variants since producing such biopharmaceuticals can be achieved faster than the synthetic mRNA vaccines. BigPharma has, in fact, promised updates to the synthetic mRNA vaccine according to the SARS-CoV-2 variants but has never introduced these to the market.

## Author contributions

GD wrote the first draft of the manuscript and all authors contributed to it and approved the final version.

